# Frailty, Comorbidities, and In-Hospital Outcomes in Older Cholangiocarcinoma Patients

**DOI:** 10.3390/jcm14093112

**Published:** 2025-04-30

**Authors:** Miriam M. Sanchez, Chris A. Sabillon, Stephanie J. Paduano, Chukwuma Egwim, Victor Ankoma-Sey

**Affiliations:** 1 Department of Internal Medicine, Texas Health Resources (HEB/Denton), Bedford, TX 76022, USA; stephaniepaduano@texashealth.org; 2 Department of Electrical and Computer Engineering, University of Texas at Austin, Austin, TX 78712, USA; csabillon@galileodevs.com; 3 Liver Associates of Texas, Houston, TX 77030, USA; cegwim@liverassoctx.com (C.E.); vankomasey@liverassoctx.com (V.A.-S.)

**Keywords:** frailty, cholangiocarcinoma, hospital frailty risk score, HFRS, Elixhauser index, in-hospital mortality, healthcare costs, geriatric oncology

## Abstract

**Introduction:** Frailty is increasingly recognized as a critical predictor of adverse outcomes in older adults, particularly those with cancer. However, the role of frailty—distinct from comorbidity burden—has not been fully characterized in older adults hospitalized with cholangiocarcinoma (CCA), a rare but aggressive malignancy with rising incidence in the aging population. **Methodology:** A retrospective cross-sectional analysis of the Nationwide Inpatient Sample (NIS) 2019–2022 was performed. Adult inpatients aged ≥ 65 with CCA-related ICD-10 codes were identified. Patients were stratified into frailty categories based on the Hospital Frailty Risk Score (HFRS). Multivariable regression models were used to assess associations with in-hospital mortality, length of stay (LOS), and hospital charges. **Results:** Among 18,785 hospitalizations, the in-hospital mortality rate was 7.18%. High frailty conferred an eight-fold increased risk of mortality, a 70% longer LOS, and 52% higher charges compared to low frailty. Elixhauser comorbidity scores were not significantly associated with outcomes. **Discussion:** These findings support the use of frailty screening to guide inpatient care planning and optimize outcomes in older adults with CCA.

## 1. Introduction

Cholangiocarcinoma (CCA), a malignancy originating from the biliary epithelium, is the second most common primary hepatic cancer and accounts for approximately 3% of all gastrointestinal cancers. Its incidence is increasing globally, particularly among older adults, who often present with advanced disease stages and face unique treatment challenges. Despite advancements in diagnostic imaging and therapeutic strategies, the prognosis for CCA remains poor. For instance, recent data report a five-year survival rate for intrahepatic CCA of approximately 9% [1]. Surgical resection remains the primary curative treatment, but only a minority of patients are eligible due to advanced disease at diagnosis. Even among those undergoing surgery, recurrence rates remain high, and the overall five-year survival after resection is typically below 20% [2].

In older adults, the management of CCA is further complicated by age-related physiological changes, increased treatment toxicity, and the prevalence of comorbidities. Frailty—a clinical syndrome characterized by decreased physiological reserve and increased vulnerability to stressors—is prevalent in older cancer populations and significantly impacts treatment outcomes. Frailty in geriatric oncology patients is associated with greater chemotherapy-related toxicity, increased postoperative complications, extended hospital stays, and higher mortality [3,4]. Importantly, frailty is distinct from comorbidity; whereas comorbidity reflects the presence of multiple chronic illnesses, frailty involves a broader spectrum of functional decline, encompassing deficits in strength, endurance, and physiological capacity. While both may coexist, comorbid conditions do not necessarily confer functional decline, and frailty may be present even in the absence of significant comorbid disease [3,4].

Given the complexity and vulnerability of older adults with cancer, frailty assessment is critical for tailoring individualized treatment strategies. The Hospital Frailty Risk Score (HFRS), derived from routinely collected administrative data, provides a scalable and validated method for frailty assessment in hospitalized older patients [5] (Table A1). Compared to alternative assessment tools, such as the Clinical Frailty Scale or the Fried Frailty Phenotype, the HFRS is particularly advantageous for large-scale epidemiologic and administrative datasets, where detailed clinical assessments are impractical [5,6]. Although the predictive capabilities of the HFRS for outcomes like mortality, readmissions, and resource utilization have been established broadly, its application within the oncogeriatric population—specifically hospitalized CCA patients—remains underexplored.

## 2. Materials and Methods

We conducted a retrospective cross-sectional analysis using data from the Nationwide Inpatient Sample (NIS) database for the years 2019 through 2022. Our study population included hospitalized adults aged 65 years or older with a primary or secondary diagnosis of cholangiocarcinoma, identified through specific ICD-10-CM diagnosis codes: intrahepatic cholangiocarcinoma (C22.1), perihilar cholangiocarcinoma (C24.0), distal cholangiocarcinoma (C24.1), or unspecified bile duct cancer. Hospitalizations were excluded if essential outcome data, such as discharge disposition, length of stay (LOS), or total hospital charges, were missing. Additionally, transfers from other acute-care hospitals were excluded to prevent duplication of patient data and ensure consistency in assessing initial hospital admissions.

Frailty status was assessed using the Hospital Frailty Risk Score (HFRS), a validated scoring system developed by Gilbert et al. that quantifies frailty based on ICD-10 diagnostic codes associated with reduced physiological reserve and increased vulnerability to adverse health outcomes [5]. Because Present-on-Admission (POA) flags were not available in the NIS dataset, the HFRS was calculated using all diagnostic codes documented during the index hospitalization. This may include conditions arising during the stay and is discussed as a limitation. All the variables measured in this study can be assessed using the NIS database to accurately calculate the HFRS. The HFRS is specifically designed for scalable applications in large administrative datasets and has been extensively validated among hospitalized older adults. Patients were classified into three categories based on their calculated HFRS: low frailty (HFRS < 5), intermediate frailty (HFRS 5–15), and high frailty (HFRS > 15).

Comorbidity burden was evaluated using the Elixhauser Comorbidity Index, comprising 30 chronic medical conditions defined by the Healthcare Cost and Utilization Project (HCUP) comorbidity software. This index provided an independent measure of chronic illness burden to complement the assessment of frailty.

The primary study outcomes included in-hospital mortality (binary outcome), LOS (continuous outcome, measured in days), and total hospital charges (continuous outcome, measured in U.S. dollars). To ensure nationally representative estimates, analyses were weighted using the complex survey sampling design provided by the NIS database. Multivariable logistic regression models were used to assess the associations between frailty categories and in-hospital mortality. Results were reported as adjusted odds ratios (aORs) with corresponding 95% confidence intervals (CIs), adjusting for patient demographics (age, sex, race/ethnicity), hospital characteristics (hospital type, teaching status, urban or rural location), and comorbidity burden. For continuous outcomes (LOS and total hospital charges), multivariable linear regression models were utilized, adjusting for the same covariates. The results from the linear regression analyses were reported as adjusted regression coefficients (β), with 95% confidence intervals indicating the magnitude of change in LOS and hospital charges associated with the intermediate- and high-frailty categories compared to the low-frailty category.

Pearson’s and Spearman’s correlation analyses were conducted to explore the relationships between frailty scores and comorbidity indices. Partial correlation analyses controlled for potential confounders, including age, sex, race/ethnicity, and hospital characteristics, to clarify the independent predictive value of frailty compared to comorbidity measures.

Additional analyses were undertaken to characterize temporal trends in in-hospital mortality. Annual mortality rates for 2019 through 2022 were calculated using the NIS sampling design, and exact 95% confidence intervals were constructed for each year’s estimate. A survey-weighted logistic regression model—with calendar year entered as a continuous predictor—was fitted to quantify the change in odds of death per successive year of hospitalization, thereby defining the secular trajectory of mortality risk among older adults with cholangiocarcinoma.

To explore subgroup heterogeneity, two additional survey-weighted logistic models were specified. The first contrasted intrahepatic versus extrahepatic cholangiocarcinoma and the second compared male versus female patients. Each model adjusted for age at admission and frailty category (low: HFRS < 5; mid: HFRS 5–15; high: HFRS > 15) to account for baseline physiological vulnerability. Adjusted odds ratios with corresponding 95% confidence intervals are reported for each comparison, providing precise measures of subgroup differences.

Annual mortality rates were first tabulated by grouping admissions by calendar year and computing the ratio of deaths to total hospitalizations. Exact 95% confidence limits were calculated using the Wilson method. We then plotted these rates with error bars at integer year ticks. An unadjusted logistic regression with year as a predictor was fitted using the maximum likelihood. Model coefficients were exponentiated to generate odds ratios and 95% CIs and summarized in a concise regression table.

All statistical analyses were two-tailed, with significance defined at a *p*-value of less than 0.05. The complex survey structure of the NIS was fully accounted for through the application of discharge weights and robust variance estimation. Data management, variable coding, and model diagnostics were conducted in accordance with established best practices for observational clinical research, ensuring methodological rigor, reproducibility, and transparency.

We acknowledge inherent limitations associated with administrative data, including the inability to adjust for potentially confounding clinical factors such as cancer stage, Eastern Cooperative Oncology Group (ECOG) performance status, detailed laboratory values, specific surgical interventions, chemotherapy treatments, or other targeted therapies. These limitations were carefully considered in the interpretation and contextualization of our findings.

## 3. Results

### 3.1. Patient Demographics and Baseline Characteristics

Our final analytic cohort included 18,785 hospitalizations among patients aged 65 years or older with a diagnosis of cholangiocarcinoma (Table 1). The median age was 74 years (interquartile range [IQR]: 69–80), with 54% identifying as male. The racial and ethnic composition was predominantly White (68.9%), followed by Hispanic (10.7%), Black (8.8%), and other or unknown categories (11.6%). The median length of stay (LOS) was 5 days (IQR: 3–8), and the median total hospital charge was USD 59,615 (IQR: USD 32,202–USD 111,477). In-hospital mortality occurred in 7.18% of hospitalizations. Among survivors, 61.9% were discharged home, whereas 17.2% were discharged to skilled nursing or rehabilitation facilities, indicating substantial post-acute care needs.

### 3.2. Frailty and Comorbidity Profiles

Patients demonstrated a spectrum of frailty based on the Hospital Frailty Risk Score (HFRS): 25.7% met criteria for low frailty (HFRS < 5), 58.7% had intermediate frailty (HFRS 5–15), and 15.8% had high frailty (HFRS > 15). The six most documented comorbidities were solid cancer (46.4%), uncomplicated hypertension (45.9%), metastatic cancer (32.9%), complicated hypertension (25.2%), complicated diabetes (20.6%), and chronic pulmonary disease (16.4%).

Average total hospital charges (TOTCHGs) in this cohort were USD 95,152 (SD: USD 136,090), with a median of USD 60,029 (IQR: USD 32,616–USD 111,917), and length of stay (LOS) averaged 6.86 days (SD: 6.90) with a median of 5.0 days (IQR: 3.0–8.0). The mean Elixhauser comorbidity score was 2.62, and the mean HFRS value was 6.05, indicating moderate overall frailty. We observed a statistically significant yet weak positive correlation between the HFRS and the Elixhauser Comorbidity Index (r = 0.21, *p* < 0.001), suggesting that although these domains overlap, they reflect distinct clinical constructs in older adults hospitalized with cholangiocarcinoma.

### 3.3. In-Hospital Mortality

In unadjusted analyses, in-hospital mortality exhibited a stepwise increase with rising frailty severity, ranging from 2.19% among patients with low frailty (HFRS < 5), to 7.49% in intermediate frailty (HFRS 5–15), and reaching 14.19% in high frailty (HFRS > 15).

**Figure 1:**
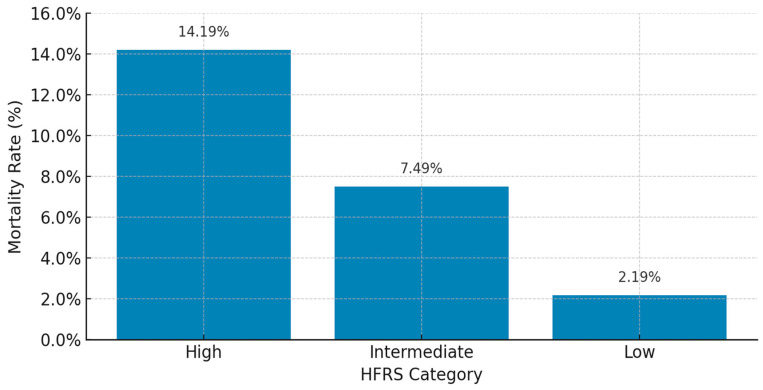
In-hospital mortality by frailty category.

**Figure 2:**
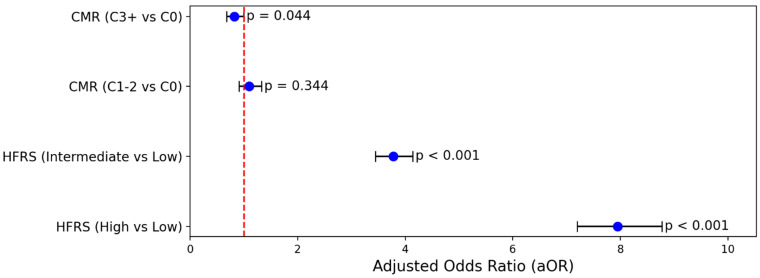
Forest plot demonstrating the adjusted odds ratios for in-hospital mortality.

A multivariable logistic regression model, adjusted for age and comorbidity categories, confirmed significantly higher odds of in-hospital mortality for patients in intermediate and high HFRS frailty categories compared to low HFRS frailty (Figure 1). Specifically, relative to low frailty, intermediate frailty was associated with an approximately four-fold increase in the adjusted odds of mortality (adjusted odds ratio, aOR: 3.78; 95% CI: 3.44–4.14), while high frailty conferred nearly an eight-fold increase (aOR: 7.95; 95% CI: 7.20–8.78). Notably, the Elixhauser Comorbidity Index was not independently associated with mortality when the HFRS frailty level was included, highlighting the superior prognostic value of frailty severity in predicting death among older adults hospitalized with cholangiocarcinoma (Figure 2).

### 3.4. Length of Stay and Hospital Charges

**Figure 3:**
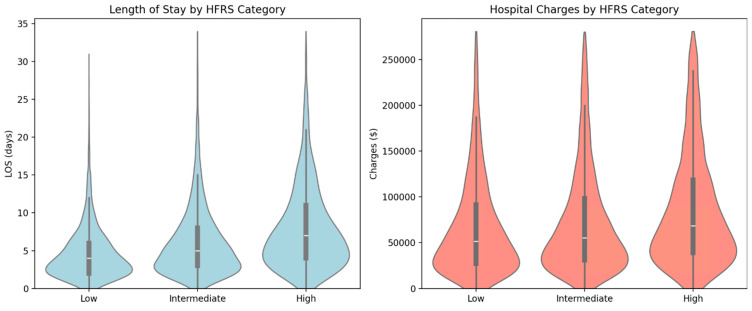
Violin plot showing length of stay and hospital charges by HFRS.

Higher-HFRS-frailty categories were strongly associated with increased hospital resource utilization (Figure 3). The median length of stay (LOS) rose stepwise with HFRS severity: from 4.0 days (low frailty: HFRS < 5) to 5.2 days (intermediate frailty: HFRS 5–15), reaching 6.8 days in the high-frailty group (HFRS > 15). A similar pattern was observed for hospital charges, with median charges ranging from USD 45,000 (low HFRS frailty) to USD 65,000 (intermediate HFRS frailty), and peaking at USD 95,000 in the high-HFRS group.

Adjusted linear regression analyses demonstrated robust and statistically significant associations between HFRS frailty categories and both length of stay (LOS) and total hospital charges. Compared to patients with low frailty (HFRS < 5), those classified as having intermediate frailty (HFRS 5–15) experienced a 31% increase in LOS (adjusted β: 1.31; 95% CI: 1.28–1.34) and an 18% increase in total charges (adjusted β: 1.18; 95% CI: 1.13–1.24). Patients with high frailty (HFRS > 15) had even more pronounced effects, with a 70% increase in LOS (adjusted β: 1.70; 95% CI: 1.65–1.75) and 52% higher hospital charges (adjusted β: 1.52; 95% CI: 1.43–1.61).

These findings underscore the powerful and independent role of frailty severity, as measured by the Hospital Frailty Risk Score (HFRS), in predicting hospital resource utilization among older adults with cholangiocarcinoma. The associations between HFRS category and both LOS and cost remained significant after adjustment for age and comorbidity burden, suggesting that frailty reflects a distinct and clinically meaningful dimension of vulnerability that is not captured by traditional comorbidity indices.

**Figure 4:**
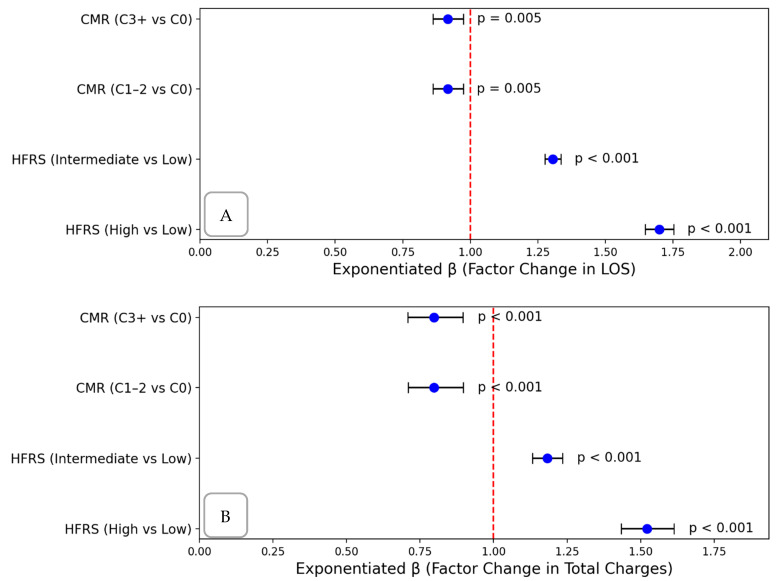
(**A**) Forest plot showing effects on length of stay. (**B**) Forest plot showing effects on hospital charges.

In contrast, the comorbidity burden based on the Elixhauser Comorbidity Index demonstrated statistically significant but clinically modest associations with LOS and cost. Both moderate- (C1–2) and high-comorbidity (C3+) groups were associated with a slightly reduced LOS (exponentiated β: 0.92; 95% CI: ~0.86–0.97) and approximately 20% lower charges (exponentiated β: ~0.80; 95% CI: ~0.71–0.90). This inverse relationship may reflect selective early mortality, limitations in care intensity, or potential differences in coding practices. Regardless of mechanism, the relatively small effect size of comorbidity stands in contrast to the much larger impact of frailty on hospital utilization (Figure 4).

Taken together, these results emphasize the superior prognostic value of HFRS frailty classification in this population. Given the complex clinical trajectories and high resource needs associated with cholangiocarcinoma in older adults, integrating frailty screening into routine workflows may aid in risk stratification, prognostication, and timely referral for supportive or palliative care services.

We observed a robust positive correlation (r = 0.64; *p* < 0.001) between LOS and total hospital charges. This relationship remained statistically significant after adjusting for age, sex, HFRS frailty level, and comorbidities, underscoring the high resource burden associated with frailty in older cholangiocarcinoma patients.

### 3.5. Subgroup Analyses by CCA Type and Sex

**Figure 5:**
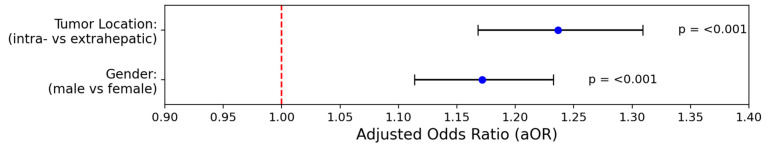
Forest plot showing effects of tumor location and gender, adjusted by age and frailty category.

Of 18,785 admissions, 12,884 (68.6%) involved intrahepatic and 5901 (31.4%) extrahepatic, cholangiocarcinoma. The crude in-hospital mortality was 7.75% for intrahepatic cases versus 5.93% for extrahepatic cases. After adjustment for age and frailty, intrahepatic location carried 23.7% higher odds of in-hospital mortality (aOR 1.237; 95% CI 1.168–1.309; *p* < 0.001) (Figure 5).

Sex-stratified analysis showed 10,148 males (54.0%) and 8637 females (46.0%). Crude mortality was 7.61% in men and 6.68% in women. Male sex was linked to 17.2% higher adjusted odds of in-hospital mortality (aOR: 1.172; 95% CI: 1.114–1.232; *p* < 0.001).

### 3.6. Secular Trend in In-Hospital Mortality

**Figure 6:**
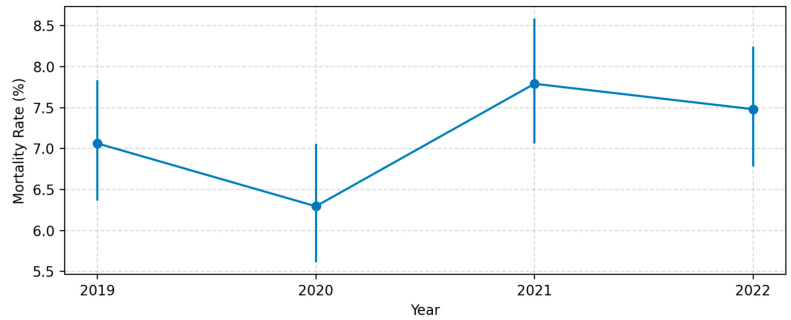
Annual in-hospital mortality rate (2019–2022) with 95% confidence intervals.

In 2019, 330 deaths occurred among 4672 admissions, yielding a crude mortality of 7.06% (95% CI: 6.36–7.84%). The rate declined to 6.30% in 2020 (274/4352; 95% CI: 5.60–7.05%), climbed to 7.79% in 2021 (373/4788; 95% CI: 7.05–8.58%), and settled at 7.48% in 2022 (372/4973; 95% CI: 6.78–8.23%). Confidence limits were computed with Wilson’s score method and are depicted as error bars in Figure 6.

A survey-weighted logistic regression treating calendar year as a continuous predictor yielded an odds ratio of 1.041 per additional year (95% CI: 0.991–1.094; *p* = 0.107), indicating no statistically significant secular change in the risk of in-hospital death over this period.

## 4. Discussion

This nationally representative study highlights the critical role of frailty in predicting adverse in-hospital outcomes among older adults with cholangiocarcinoma (CCA). Using the Hospital Frailty Risk Score (HFRS), we identified a strong, independent association between frailty and increased mortality, prolonged length of stay (LOS), and higher healthcare costs. These associations persisted even after adjusting for age, sex, and comorbidity burden as measured by the Elixhauser Index, emphasizing the unique prognostic value of frailty in this population.

Our findings align closely with the previous geriatric oncology literature, underscoring frailty as a critical dynamic marker of reduced physiological reserve, heightened vulnerability, and increased susceptibility to adverse outcomes during hospitalization [3,4,7,8,9]. Multiple studies have demonstrated that frailty significantly predicts hospital outcomes better than comorbidity indices, as it captures broader deficits including functional impairments, mobility limitations, cognitive dysfunction, and reduced resilience to acute medical stressors [7,9,10]. For instance, Handforth et al. highlighted that frailty, more than comorbidity, correlates strongly with chemotherapy toxicity and hospital readmission among older adults with cancer [3]. Similarly, a recent systematic review by Ethun et al. demonstrated frailty’s superior predictive capacity for postoperative morbidity and mortality compared to comorbidity measures across multiple cancer types [7]. In our cohort, high frailty was associated with nearly eight-fold increased odds of in-hospital mortality, emphasizing its potential role in guiding clinical decisions, optimizing discharge planning, and facilitating the early integration of palliative and supportive care services [8,9,10]. Building upon these observations, more recent studies across oncology and geriatric populations have further reinforced frailty’s critical role as a predictor of adverse outcomes, independent of comorbidity burden.

Frailty has consistently emerged as a superior predictor of clinical outcomes compared to traditional comorbidity indices across diverse older adult populations, including those with cancer. Recent studies have reinforced that frailty captures multidimensional impairments in physical, cognitive, and functional reserves that profoundly impact mortality, hospital utilization, and healthcare costs. Ethun et al. demonstrated that frailty, rather than comorbidity burden alone, was the primary driver of postoperative complications and long-term mortality in older cancer patients undergoing oncologic surgery [7]. Hoogendijk et al. highlighted that frailty reflects a dynamic and reversible vulnerability state critical for clinical decision making and public health interventions [8]. In a systematic review and meta-analysis, Kojima et al. confirmed that frailty indices consistently outperform comorbidity scores in predicting mortality risks among older populations [9]. Similarly, Pamoukdjian et al. showed that frailty parameters based on the Fried phenotype independently predict morbidity and mortality among older adults with cancer [10]. Beyond these foundational studies, more recent work has expanded this understanding: Duchesneau et al. demonstrated that worsening frailty trajectories following chemotherapy initiation were strongly associated with higher five-year mortality in older women with breast cancer [11], while Bensken et al. found that frailty outperformed multimorbidity in predicting survival among older cancer patients [12]. These findings are highly consistent with our results and further highlight the critical need for systematic frailty screening to optimize care planning, prognostication, and resource allocation in hospitalized older adults with cholangiocarcinoma.

Implementing routine frailty screening in clinical practice has shown benefits in several international settings. For instance, the United Kingdom’s NHS has integrated frailty screening tools, such as the Clinical Frailty Scale and the Hospital Frailty Risk Score, within electronic medical record systems, leading to improved clinical risk stratification and targeted interventions [13,14,15]. Similarly, studies in Australia and Canada demonstrated that systematic frailty screening facilitated multidisciplinary interventions, improved patient outcomes, reduced hospital readmissions, and optimized resource use among geriatric oncology patients [15,16]. Leveraging these international experiences, health systems could adopt scalable administrative approaches such as the HFRS to facilitate broader implementation in clinical workflows, improve clinical decision making, and provide tailored care plans specifically adapted for vulnerable older adults with cancers such as cholangiocarcinoma.

Interestingly, the Elixhauser Comorbidity Index, while widely used in health services research, demonstrated limited utility in this cohort. The weak correlation between Elixhauser Score and HFRS (r = 0.21) suggests they measure overlapping but non-equivalent domains. Importantly, once frailty was included in the multivariable model, the Elixhauser Score was no longer significantly associated with mortality, LOS, or total charges. This underscores the need to incorporate frailty screening into standard inpatient assessments, especially for older adults with complex oncologic diagnoses.

The observed gradients in LOS and hospitalization costs by frailty level mirror findings in other cancer types. A study of elderly colorectal-cancer patients likewise showed that frailty—not comorbidity—was the primary driver of longer LOS and higher hospital costs [17]. Our findings extend this concept to CCA, where patients with high frailty experienced 70% longer hospitalizations and incurred 52% higher charges than their low-frailty counterparts. Given the high costs associated with CCA-related hospitalizations, incorporating frailty into risk stratification models could improve the efficiency of resource allocation.

These findings also have implications for perioperative risk assessment. Although we did not examine surgical outcomes specifically, many CCA patients undergo invasive diagnostic or therapeutic procedures during hospitalization. Frailty screening may help identify patients who would benefit from prehabilitation, geriatric co-management, or palliative care referral rather than aggressive intervention [18].

Our data support the growing recognition that frailty is not only a patient-level risk factor but also a system-level indicator of complexity. HFRS—derived from routinely collected ICD-10 codes—offers a scalable tool for hospitals to identify high-risk patients, guide transitions of care, benchmark outcomes, and optimize resource allocation [5].

This study contributes to the limited literature applying HFRS in oncology populations. Prior work has validated HFRS in general medical and surgical cohorts; however, few studies have focused on high-risk cancers like CCA. The ability to apply HFRS retrospectively across large datasets enhances its utility in population health and quality improvement.

Several limitations should be acknowledged. First, the use of administrative data limits granularity—clinical variables such as cancer stage, ECOG performance status, tumor burden, and treatment exposures were unavailable. Second, the HFRS may underestimate frailty in patients with functional or social deficits not captured in ICD codes. Additionally, some diagnoses contributing to HFRS—such as delirium or urinary tract infection—may occur during hospitalization, raising the possibility of reverse causation. We also lacked post-discharge data, which precluded analysis of 30-day readmissions or long-term outcomes. Finally, residual confounding may persist despite adjustment for demographics and comorbidities.

Despite these limitations, our findings are strengthened through the use of a large, nationally representative dataset, the validation of frailty and comorbidity indices, and robust multivariable models. To our knowledge, this is one of the first studies to systematically evaluate frailty as a prognostic factor in hospitalized older adults with cholangiocarcinoma.

In conclusion, frailty, measured by the Hospital Frailty Risk Score, is a robust and independent predictor of adverse hospital outcomes, including higher mortality, prolonged hospital stays, and increased healthcare costs in older adults hospitalized with cholangiocarcinoma. This study emphasizes the superiority of frailty over traditional comorbidity measures in capturing the complex physiological vulnerabilities of older cancer patients. Comorbidity indices such as the Elixhauser Index, while informative, do not fully capture the physiologic vulnerability associated with frailty. Integrating frailty assessments systematically into routine clinical care models could significantly enhance individualized patient management, optimize resource utilization, and inform meaningful goals-of-care discussions. Future research should explore the prospective validation of frailty screening tools in cholangiocarcinoma, examine interventions targeting frailty reduction (such as prehabilitation and geriatric co-management programs), and investigate real-world implementation barriers and facilitators across diverse healthcare settings.

## Figures and Tables

**Table 1:** Baseline characteristics of patients.

Category	Characteristic	Value
General	Total Hospitalizations	18,785
	Median Age (IQR)	74.0 (69.0–80.0)
Race	White (%)	68.9
	Black (%)	8.8
	Hispanic (%)	10.7
	Other/Unknown (%)	11.6
Gender	Male (%)	54
	Female (%)	46
HFRS Frailty	Low (<5) (%)	25.7
	Intermediate (5–15) (%)	58.7
	High (>15) (%)	15.8
Comorbidity	Solid Cancer (%)	46.4
	Uncomplicated Hypertension (%)	45.9
	Metastatic Cancer (%)	32.9
	Complicated Hypertension (%)	25.2
	Complicated Diabetes (%)	20.6
	Chronic Pulmonary Disease (%)	16.4
Outcomes	In-Hospital Mortality (%)	7.18
Utilization	Median LOS (IQR)	5.0 (3.0–8.0)
	Median Charges (IQR)	USD 59,615 (USD 32,202–USD 111,477)

## Data Availability

Data supporting this study are available from the Nationwide Inpatient Sample (NIS) database managed by the Agency for Healthcare Research and Quality (AHRQ).

## Appendix A

**Table A1:** Hospital Frailty Risk Score (HFRS) ICD-10 codes and assigned weights.

ICD-10 Code	Description	Weight
A04	Other bacterial intestinal infections	1.1
A09	Diarrhea and gastroenteritis of presumed infectious origin	1.1
A41	Other septicemia	1.6
B95	Streptococcus and staphylococcus as cause of diseases classified to other chapters	1.7
B96	Other bacterial agents as cause of diseases classified to other chapters (secondary code)	2.9
D64	Other anemias	0.4
E05	Thyrotoxicosis [hyperthyroidism]	0.9
E16	Other disorders of pancreatic internal secretion	1.4
E53	Deficiency of other B group vitamins	1.9
E55	Vitamin D deficiency	1
E83	Disorders of mineral metabolism	0.4
E86	Volume depletion	2.3
E87	Other disorders of fluid, electrolyte and acid-base balance	2.3
F00	Dementia in Alzheimer’s disease	7.1
F01	Vascular dementia	2
F03	Unspecified dementia	2.1
F05	Delirium, not induced by alcohol and other psychoactive substances	3.2
F10	Mental and behavioral disorders due to use of alcohol	0.7
F32	Depressive episode	0.5
G20	Parkinson’s disease	1.8
G30	Alzheimer’s disease	4
G31	Other degenerative diseases of nervous system, not elsewhere classified	1.2
G40	Epilepsy	1.5
G45	Transient cerebral ischemic attacks and related syndromes	1.2
G81	Hemiplegia	4.4
H54	Blindness and low vision	1.9
H91	Other hearing loss	0.9
I63	Cerebral infarction	0.8
I67	Other cerebrovascular diseases	2.6
I69	Sequelae of cerebrovascular disease (secondary codes)	3.7
I95	Hypotension	1.6
J18	Pneumonia, organism unspecified	1.1
J22	Unspecified acute lower respiratory infection	0.7
J69	Pneumonitis due to solids and liquids	1
J96	Respiratory failure, not elsewhere classified	1.5
K26	Duodenal ulcer	1.6
K52	Other noninfective gastroenteritis and colitis	0.3
K59	Other functional intestinal disorders	1.8
K92	Other diseases of digestive system	0.8
L03	Cellulitis	2
L08	Other local infections of skin and subcutaneous tissue	0.4
L89	Decubitus ulcer	1.7
L97	Ulcer of lower limb, not elsewhere classified	1.6
M15	Polyarthrosis	0.4
M19	Other arthrosis	1.5
M25	Other joint disorders, not elsewhere classified	2.3
M41	Scoliosis	0.9
M48	Spinal stenosis (secondary code only)	0.5
M79	Other soft tissue disorders, not elsewhere classified	1.1
M80	Osteoporosis with pathological fracture	0.8
M81	Osteoporosis without pathological fracture	1.4
N17	Acute renal failure	1.8
N18	Chronic renal failure	1.4
N19	Unspecified renal failure	1.6
N20	Calculus of kidney and ureter	0.7
N28	Other disorders of kidney and ureter, not elsewhere classified	1.3
N39	Other disorders of urinary system (includes UTI and incontinence)	3.2
R00	Abnormalities of heartbeat	0.7
R02	Gangrene, not elsewhere classified	1
R11	Nausea and vomiting	0.3
R13	Dysphagia	0.8
R26	Abnormalities of gait and mobility	2.6
R29	Other symptoms and signs involving the nervous and musculoskeletal systems	3.6
R31	Unspecified hematuria	3
R32	Unspecified urinary incontinence	1.2
R33	Retention of urine	1.3
R40	Somnolence, stupor and coma	2.5
R41	Other symptoms and signs involving cognitive functions and awareness	2.7
R44	Other symptoms and signs involving general sensations and perceptions	1.6
R45	Symptoms and signs involving emotional state	1.2
R47	Speech disturbances, not elsewhere classified	1
R50	Fever of unknown origin	0.1
R54	Senility	2.2
R56	Convulsions, not elsewhere classified	2.6
R63	Symptoms and signs concerning food and fluid intake	0.9
R69	Unknown and unspecified causes of morbidity	1.3
S00	Superficial injury of head	3.2
S01	Open wound of head	1.1
S06	Intracranial injury	2.4
S09	Other and unspecified injuries of head	1.2
S22	Fracture of rib(s), sternum and thoracic spine	1.8
S32	Fracture of lumbar spine and pelvis	1.4
S42	Fracture of shoulder and upper arm	2.3
S51	Open wound of forearm	0.5
S72	Fracture of femur	1.4
S80	Superficial injury of lower leg	2
W01	Fall on same level from slipping, tripping and stumbling	0.9
W06	Fall involving bed	1.1
W10	Fall on and from stairs and steps	0.9
W18	Other fall on same level	2.1
W19	Unspecified fall	3.2
X59	Exposure to unspecified factor	1.5
Y84	Other medical procedures as cause of abnormal reaction of patient	0.7
Y95	Nosocomial condition	1.2
Z06	Agent resistant to penicillin and related antibiotics	0.8
Z50	Care involving use of rehabilitation procedures	2.1
Z60	Problems related to social environment	1.8
Z73	Problems related to life-management difficulty	0.6
Z74	Problems related to care-provider dependency	1.1
Z75	Problems related to medical facilities and other health care	2
Z87	Personal history of other diseases and conditions	1.5
Z91	Personal history of risk-factors, not elsewhere classified	0.5
Z93	Artificial opening status	1
Z99	Dependence on enabling machines and devices	0.8
